# Photoinduced Enhancement of Photoluminescence of Colloidal II-VI Nanocrystals in Polymer Matrices

**DOI:** 10.3390/nano10122565

**Published:** 2020-12-21

**Authors:** Volodymyr Dzhagan, Oleksandr Stroyuk, Oleksandra Raievska, Oksana Isaieva, Olga Kapush, Oleksandr Selyshchev, Volodymyr Yukhymchuk, Mykhailo Valakh, Dietrich R. T. Zahn

**Affiliations:** 1 V. Lashkaryov Institute of Semiconductors Physics, National Academy of Sciences of Ukraine, 01601 Kyiv, Ukraine; oksanka.isayeva@gmail.com (O.I.); savchuk-olja@ukr.net (O.K.); yukhym@isp.kiev.ua (V.Y.); valakh@isp.kiev.ua (M.V.); 2 Department of Physics, Taras Shevchenko National University of Kyiv, 64 Volodymyrs’ka St., 01601 Kyiv, Ukraine; 3 Forschungszentrum Julich GmbH, Helmholtz-Institut Erlangen Nürnberg für Erneuerbare Energien (HI ERN), Immerwahrstr. 2, 91058 Erlangen, Germany; o.stroyuk@fz-juelich.de; 4 Semiconductor Physics, Chemnitz University of Technology, D-09107 Chemnitz, Germany; oleksandra.raievska@physik.tu-chemnitz.de (O.R.); oleksandr.selyshchev@physik.tu-chemnitz.de (O.S.); zahn@physik.tu-chemnitz.de (D.R.T.Z.); 5 Center for Materials, Architectures, and Integration of Nanomembranes (MAIN), Chemnitz University of Technology, 09107 Chemnitz, Germany; 6 L.V. Pysarzhevsky Institute of Physical Chemistry, National Academy of Sciences of Ukraine, 03028 Kyiv, Ukraine

**Keywords:** colloidal quantum dots, CdSe/CdS, CdSe/ZnS, photo-activation, photo-brightening

## Abstract

The environment strongly affects both the fundamental physical properties of semiconductor nanocrystals (NCs) and their functionality. Embedding NCs in polymer matrices is an efficient way to create a desirable NC environment needed for tailoring the NC properties and protecting NCs from adverse environmental factors. Luminescent NCs in optically transparent polymers have been investigated due to their perspective applications in photonics and bio-imaging. Here, we report on the manifestations of photo-induced enhancement of photoluminescence (PL) of aqueous colloidal NCs embedded in water-soluble polymers. Based on the comparison of results obtained on bare and core/shell NCs, NCs of different compounds (CdSe, CdTe, ZnO) as well as different embedding polymers, we conclude on the most probable mechanism of the photoenhancement for these sorts of systems. Contrary to photoenhancement observed earlier as a result of surface photocorrosion, we do not observe any change in peak position and width of the excitonic PL. Therefore, we suggest that the saturation of trap states by accumulated photo-excited charges plays a key role in the observed enhancement of the radiative recombination. This suggestion is supported by the unique temperature dependence of the trap PL band as well as by power-dependent PL measurement.

## 1. Introduction

Semiconductor nanocrystals (NCs) and quantum dots (QDs) prepared by colloidal syntheses represent an ever-growing area of fundamental research that envisages numerous applications [1,2]. NCs with strong absorption over the solar irradiation range were developed for new generation solar cells [3]. Due to the large surface-to-volume ratio, NCs are efficient for photocatalytic conversion, which can be further improved by surface functionalization and by tuning the absolute position of the conduction and valence band edges by NC size dependent quantum confinement [4]. Size dependent photoluminescence (PL) and electroluminescence are the inherent properties of NCs that are promising for novel lighting [5] and bioimaging applications [6]. Even though the recent trend towards so-called “greener” NC materials, such as Ag-In-S and Ag-In-S/ZnS [7], Cu_2_ZnSnS_4_ [8], Cu_3_SnS_4_ [9], Cu-In-S(e) [10,11,12] is observed, II-VI NCs remain a “benchmark” model system for the investigation of the confinement-related phenomena [13], surface chemistry and physics [14], and electronic interactions of strongly confined semiconductor NC with excited molecular [15] and plasmonic [16,17,18] species. Incorporation of semiconductor NCs in polymer matrices is a major step in a big segment of applications exploiting the optical/luminescent properties of NCs [1,5]. 

A vital aspect of the luminescent properties of NCs is the predictable behavior of the PL emission with time [19]. Most applications demand stability of the PL intensity, peak position, and spectral line shape. Robust NCs are required for LEDs, lasing, and optical imaging. At the same time, numerous other potential NC applications rely on a variation of the PL intensity or shift of the PL maximum (color change) in response to certain stimuli [20]. For instance, brightening or quenching of the PL emission is the basics of optical readout of chemical sensors [21] and (nano)thermometers [22]. Therefore, the investigation of the temporal behavior of the PL spectra of QDs, in particular in application-relevant environments, such as water solutions or polymer matrices, is vitally important. There are studies of the PL spectra of colloidal II-VI NCs under a prolonged illumination in solutions [23,24,25,26,27,28], dried on substrates [29,30,31], and embedded in polymers [23,24,32,33,34,35]. The studies revealed a puzzling variety of trends in intensity and spectral changes, indicating multifaceted physical and chemical processes underlying the apparent PL photoenhancement, which differ significantly between particular studies. In most cases, the photoenhancement was studied for NCs possessing only excitonic PL band and was accompanied by a blue-shift of the PL band [23,34], indicative of photocorrosion of the NC (surface).

In this work, we investigated the evolution of the PL spectra of different types of II-VI NCs, embedded in several polymer matrices, under continuous above-bandgap illumination with laser light. The analysis of this evolution gives not only information about the durability of certain NC/polymer composites, but also helps to shed light on the structure of the NC/polymer interface and the nature of the observed PL emission. Temperature and excitation power dependences of the PL spectra were also studied, as they provide additional information on the redistribution of charge carriers between the electronic states. We investigated two types of samples: (i) NCs synthesized in the presence of polymers acting as stabilizers and (ii) NCs synthesized (ex situ) using other stabilizers/ligands and subsequently embedded into a polymer.

## 2. Materials and Methods 

The synthesis and basic characterization (UV-vis absorption, X-ray diffraction, electron microscopy etc.) of colloidal NCs used in this work was reported by us in detail previously. In particular, the synthesis of in situ polymer-stabilized CdSe, CdSe/CdS, and CdSe/ZnS NCs and the preparation of NC/polymer films was described in Reference [36]. Synthesis of CdTe NCs stabilized with thioglycolic acid (TGA) in aqueous solution was reported in References [37,38] and of acetate-stabilized ZnO NCs in dimethyl sulfoxide (DMSO) in Reference [39]. For CdTe and ZnO NCs, NC/polymer films were prepared by mixing of the initial NC solution with different amounts of aqueous solution (approx. 5 wt%) of the corresponding polymer and drying the solvent at room temperature under a hood. 

The PL spectra were registered using photoexcitation with 458 and 514.5 nm lines of an Ar-ion laser, 514.7 nm diode pumped solid state (DPSS) laser (Cobolt), or 325 nm He-Cd laser line and recorded at a spectral resolution better than 1 nm using a single spectrometer of Dilor XY or LabRam HR800 micro-Raman systems. The laser power on the sample was in the range of 0.1 to 100 W/cm^2^. Temperature-dependent PL measurements for ZnO NCs (80–290 K) were performed using a Linkam Stage THMS-600 micro-chamber (Linkam Scientific Instruments Ltd., Waterfield, United Kingdom), for CdSe and CdSe/ZnS NCs (10–300 K) using closed-cycle helium cryostat (Oxford Instruments, Wiesbaden, Germany). The samples were mounted directly on the “cold finger” using a thermally conductive clue. Spectra were acquired in 10 °C steps, with 3 min waiting time applied upon reaching each set temperature, to ensure the thermal equilibrium was reached between the sample and the thermocouple measuring the temperature (on the “cold finder” aside the sample). The time-dependent evolution of the PL spectra was studied by recording sequences of the PL spectra in certain time intervals (indicated in the manuscript for each of the relevant figures). The total time required to accumulate and save the spectrum was only 1–2 s, so it is included in the corresponding indicated time intervals.

## 3. Results and Discussion

### 3.1. PL Photoenhancement of Polymer-Stabilized Cdse and Core/Shell NCs

**Figure 1:**
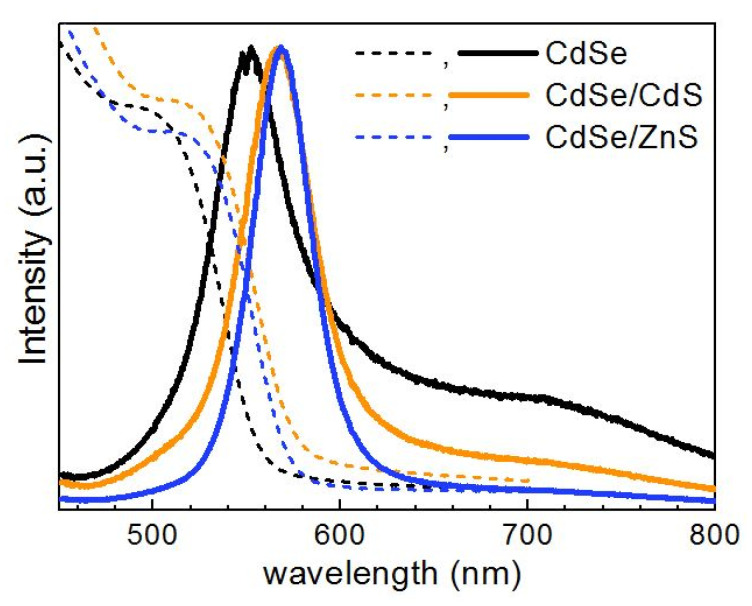
Representative optical absorption (dashed lines) and photoluminescence (PL) (solid lines, λ_exc_ = 325 nm) of a series of about 3nm CdSe NCs and 4 nm core/shell NCs in gelatin matrix.

In this section we describe the results obtained for NCs that were synthesized directly in water solutions of polymers, i.e., the polymer served as a stabilizer of NCs in the colloidal solution. Subsequently, the formation of the NC/polymer composite films was performed by depositing the as-synthesized solution on a substrate and leaving it drying naturally. The UV-vis absorption and PL spectra of the CdSe, CdSe/CdS, and CdSe/ZnS NCs discussed in this section are shown in Figure 1.

**Figure 2:**
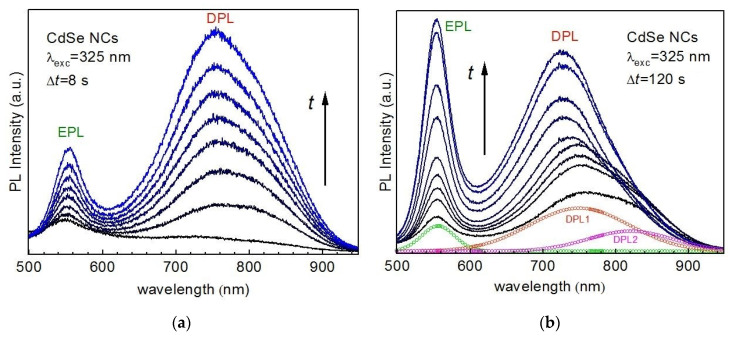
Evolution (from black to blue) of the room-temperature PL spectra (λ_exc_ = 325 nm, 0.1 W/cm^2^) of CdSe NCs in a gelatin matrix at different time-scales, with the periods between the spectra equal to 8 s (**a**) and 120 s (**b**). EPL and DPL denotes, respectively, the excitonic emission band and the trap/defect-related band. A representative deconvolution of the latter into two components, DPL1 and DPL2, is shown for the bottom spectrum in (**b**).

Figure 2 shows the temporal evolution of the PL spectra of CdSe NCs in a gelatin matrix (CdSe-gel). Similar to numerous previous reports on CdSe and other II-VI NCs [40,41,42,43,44], the spectrum consists of a relatively sharp band in the green spectral range (~555 nm), attributed to band-edge or excitonic emission (denoted as EPL in this paper), and a broad surface or trap related PL band in the red (~750–800 nm, denoted as DPL in this paper). 

The integrity of the DPL band is subject of ongoing discussion in the community [13,45,46,47]. In our spectra, this band may be regarded as consisting of one component peaked at about 750 nm (denoted as DPL1) and another one at 800–820 nm (DPL2). Under illumination with 325 nm laser light (~0.1 W/cm^2^), both PL bands gain in intensity (Figure 2). First, at the timescale of seconds to tens of seconds, the DPL bands grow faster than the EPL one (Figure 2a). At longer illumination times, tens to hundreds of seconds, EPL starts to restore its higher relative intensity to DPL (Figure 2b). 

**Figure 3:**
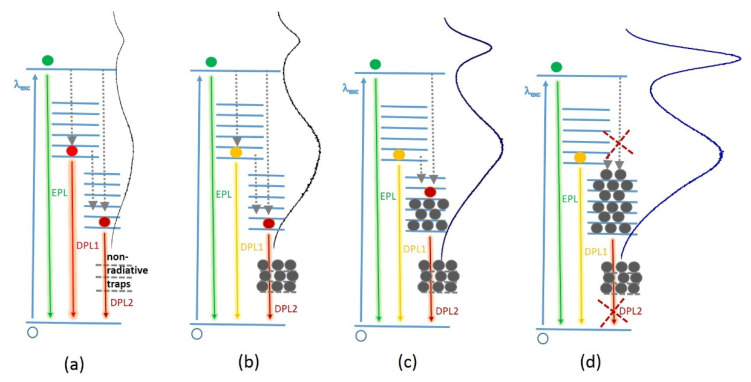
A schematic representing the mechanism proposed in this work to interpret the photoinduced changes (photoenhancement) in the PL spectra of NCs in a polymer matrix. The dotted lines denote non-radiative transitions of the photoexcited carrier to the trap states (trapping). Charge carriers that fill the states and block PL emission from them are depictured in grey. The charge carriers in three other colors denote those available for different recombination mechanisms (indicated by an arrow of the same color): excitonic or near-bandgap PL (EPL, green); defect related PL bands DPL1 (yellow) and DPL2 (red). The evolution of the energy level filling upon NC photoexcitation is as follows: (**a**) initially both radiative and non-radiative traps are empty; (**b**) filling of the non-radiative traps and concomitant increase of an overall PL intensity; (**c**) partial filling of the radiative trap levels underlying DPL2 band leads to blue shift of the whole DPL band; (**d**) complete filling of the DPL2 trap levels and disappearance of DPL2 contribution from the spectrum.

From the unchanged position of the EPL band, we can conclude that no noticeable photooxidation (photocorrosion) takes place the under given illumination conditions. This is different from numerous previous results on II-VI NCs in solutions or polymers [23,32,33,34,35], which revealed a blue shift of the optical spectra, indicative of the NC size reduction during exposure to light. Notably, along with restoring the EPL/DPL ratio at longer exposure times, the DPL bands show a blue shift and narrowing, which can be understood as a result of intensity redistribution between the longer- (DPL2) and shorter-wavelength (DPL1) components of the DPL band. We note, that the interpretation of the broad and often asymmetric DPL band of II-VI and other NCs as a superposition of two or more PL components related with different recombination routes was also proposed in other studies [13,48,49]. The spectral behavior of the apparently complex DPL band in Figure 2b could be explained by assuming an elimination of defects or other trap states mediating the DPL2 recombination, as a result of photoannealing or photocorrosion. However, this is excluded in our case because of the unaffected EPL peak position and FWHM. Therefore, we suggest an alternative explanation that assumes filling of gap states mediating DPL2 with electrons during prolonged illumination with laser light. The saturation of the gap states precludes radiative transition of photoexcited electrons into (or from) these states, as schematically shown in Figure 3. For the model to explain the initial enhancement of the PL spectrum as a whole (i.e., both EPL and DPL), we assume that neutralization of non-radiative traps occurs during illumination by filling them with photoexcited charge carriers (Figure 3a,b). Subsequent filling of the radiative traps is supposed to cause blue shift and narrowing of the entire DPL band due to suppression of the DPL2 component (Figure 3c,d).

**Figure 4:**
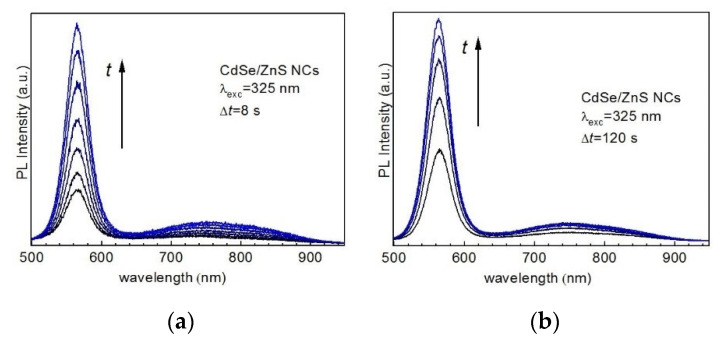
Evolution (from black to blue) of the room-temperature PL spectra (λ_exc_ = 325 nm, 0.1 W/cm^2^) of CdSe/ZnS NCs in gelatin matrix, with the periods between the spectra equal to 8 s (**a**) and 120 s (**b**).

The PL spectra of CdSe/ZnS core/shell NCs also reveal noticeable photoenhancement (Figure 4). However, the gain in intensity occurs predominantly for EPL, while the DPL reveals only a moderate increase in intensity. The EPL band gets enhanced at constant peak position and width, similarly to bare CdSe NCs. 

Therefore, our observations for CdSe/ZnS NCs are in good agreement with the enhancement mechanism suggested above for CdSe NCs, which does not involve photooxidation or photoannealing as the main factor. The ZnS (or other larger-bandgap material) shell is known to provide a higher EPL/DPL intensity ratio and a higher total PL intensity due to the passivation of both non-radiative and radiative surface traps [50,51]. In addition, the shell with a larger bandgap than that of the core creates an energy barrier for both electrons and holes, precluding them from escaping into the environment (matrix, solvent) and thus further increasing the rate of their radiative recombination [50]. Indeed, we observe a larger EPL/DPL intensity ratio in CdSe/ZnS (Figure 4) compared to CdSe NCs (Figure 2). Therefore, the rate of increasing EPL/DPL intensity ratio under illumination being higher for CdSe/ZnS NCs (Figure 4) than for CdSe NCs (Figure 2) can be explained by a lower concentration of both radiative and non-radiative traps in the core/shell sample.

**Figure 5:**
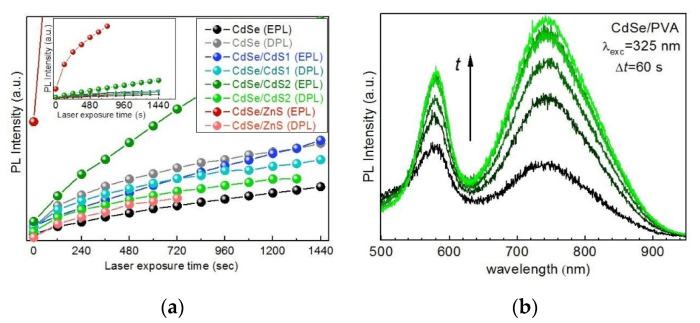
(**a**) Time evolution of the room-temperature PL intensity (λ_exc_ = 325 nm) of CdSe, CdSe/CdS, and CdSe/ZnS NCs in gelatin matrix. The integrated PL intensity was derived from the time-resolved series of spectra like those in the Figure 2 and Figure 4. The inset shows the same dependences but in a full intensity range of the EPL of CdSe/ZnS (**b**) Evolution (from black to green) of the room-temperature PL spectra (λ_exc_ = 325 nm) of CdSe NCs in PVA matrix.

For CdSe/CdS samples (spectra not shown, Figure 5a summarizes the photoenhancement behavior of all three types of NC samples in gelatin matrix), which had both the absolute PL intensity and the EPL/DPL ratio intermediate between those of the CdSe and CdSe/ZnS NCs analyzed above, an intermediate trend was observed in terms of photoenhancement, thus further confirming the viability of the underlying mechanism proposed above. 

The determinant role of the surface and stabilizing agent in the PL properties of colloidal NCs is generally known [50]. For NCs synthesized using polymers as stabilizers, with subsequent transfer of NCs into polymer matrix by simple evaporation of the solvent (water in our case), the type of polymer is expected to have an effect not only on the PL spectrum and quantum yield but also on the PL behavior under illumination. Indeed, CdSe NCs synthesized in a polyvinyl alcohol (PVA) matrix (Figure 5b) reveal a weaker photoenhancement compared to that of the CdSe-gel NCs analyzed above. A possible reason for that can be the relatively higher oxygen permeability of PVA films, with oxygen being an efficient scavenger of (photoexcited) electrons [4]. Additionally, recent detailed studies of similarly synthesized CdS/PVA NCs assumed an important role of the electronic trap states in the polymer in the PL properties of the NC [15,52,53,54].

### 3.2. Effect of λ_exc_

**Figure 6:**
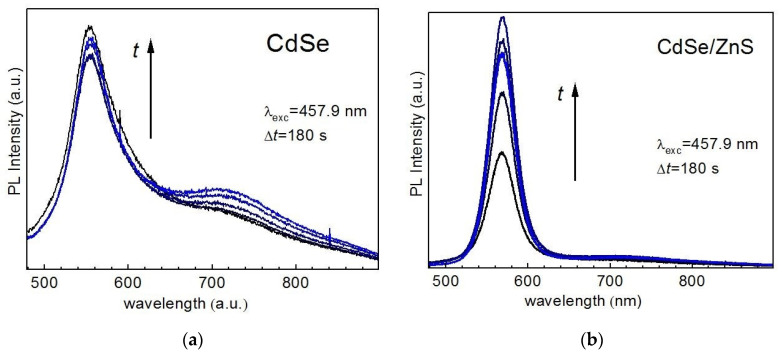
(**a**) Time evolution (from black to blue) of the PL intensity of CdSe (**a**) and CdSe/ZnS (**b**) NCs in gelatin matrix at λ_exc_ = 457.9 nm (0.1 W/cm^2^).

Even though showing a less pronounced effect than that at UV excitation (described above), the photoenhancement also takes place at visible λ_exc_. Moreover, it shows qualitatively the same trend—the total PL intensity growth is stronger for core/shell than for bare NCs, and core NCs show stronger enhancement of DPL, while EPL is predominantly enhanced in core shell NCs (Figure 6).

### 3.3. Temperature Dependence of PL Spectra

**Figure 7:**
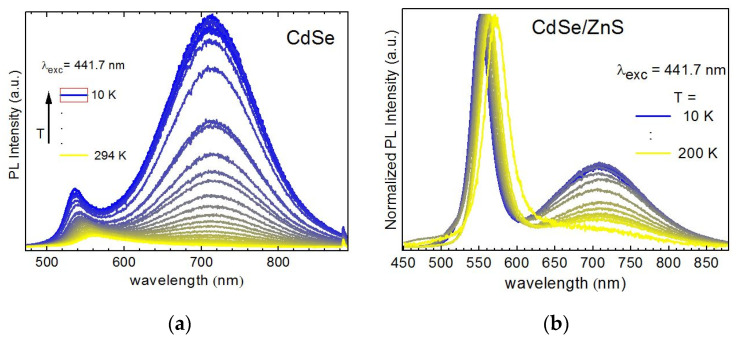
Temperature dependence of the PL spectra of CdSe (**a**) and CdSe/ZnS (**b**) NCs in gelatin matrix (λ_exc_ = 441.7 nm, 0.1 W/cm^2^).

The investigation of the temperature dependence of the shape and intensity of PL bands is a very informative tool of obtaining information about the (re)distribution of charge carriers over the energy levels in bulk and NC semiconductors. In particular, a different evolution of the PL spectra of bare CdSe (Figure 7a) and core/shell CdSe/ZnS NCs (Figure 7b) with temperature provides additional arguments in support of the PL photoenhancement mechanism proposed and discussed above. Besides the common blue shift and narrowing of EPL, inherent to PL recombination from band states of a crystalline semiconductor, a different evolution of EPL/DPL intensity ratio is observed for the two types of NCs. Bare CdSe NCs reveal a much stronger relative enhancement of the DPL band than core/shell NCs (Figure 7). This trend can be well understood in view of a larger concentration of radiative traps on the surface of bare CdSe NCs.

**Figure 8:**
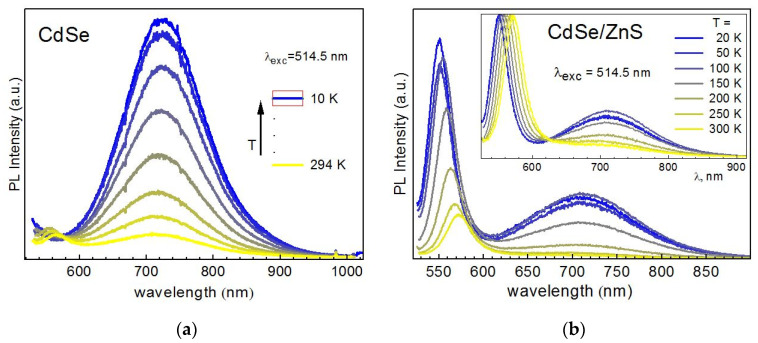
Temperature dependence of the PL spectra of CdSe (**a**) and CdSe/ZnS (**b**) NCs in gelatin matrix (λ_exc_ = 514.5 nm, 0.1 W/cm^2^). Inset in (**b**) shows normalized curves.

Qualitatively, the same trend was found in temperature dependence obtained with λ_exc_ = 514.5 nm (Figure 8), confirming the general nature of the phenomenon observed. One distinct feature is though observed at λ_exc_ = 514.5 nm for CdSe NCs. In particular, the EPL band gradually disappears from the spectrum at certain temperatures (Figure 8a). This fact is rather straightforward to understand—when the energy of the bandgap becomes larger than the energy of the excitation quanta, the interband excitation of electrons is not possible anymore. Surprisingly, this situation does not affect the DPL band—its intensity continues to grow steadily even after the disappearance of the EPL band (Figure 8a). This experimental observation is very important for understanding the electronic processes behind the optical properties of NCs. In particular, it indicates that the “supply” of the charge carriers to the electronic states responsible for DPL emission does not necessarily involve the step of interband excitation/absorption but may apparently occur “directly” (via direct subbandgap photoexcitation). In view of the rather high intensity of the DPL emission, this transfer of photoexcited charges or energy to DPL emitting states must be very efficient. A relevant model involved for explaining such high efficiency can be the self-trapped exciton (STE) model proposed recently for the interpretation of highly efficient broad-band PL of ultrasmall II-VI [13,47] and I-III-VI [10,47] colloidal NCs. This model assumes a strong electron-optical phonon coupling (EPC) of the photoexcited charges in very small NCs and provides also a reasonable explanation for an anomalous temperature dependence of their broad-band PL spectra [47]. 

**Figure 9:**
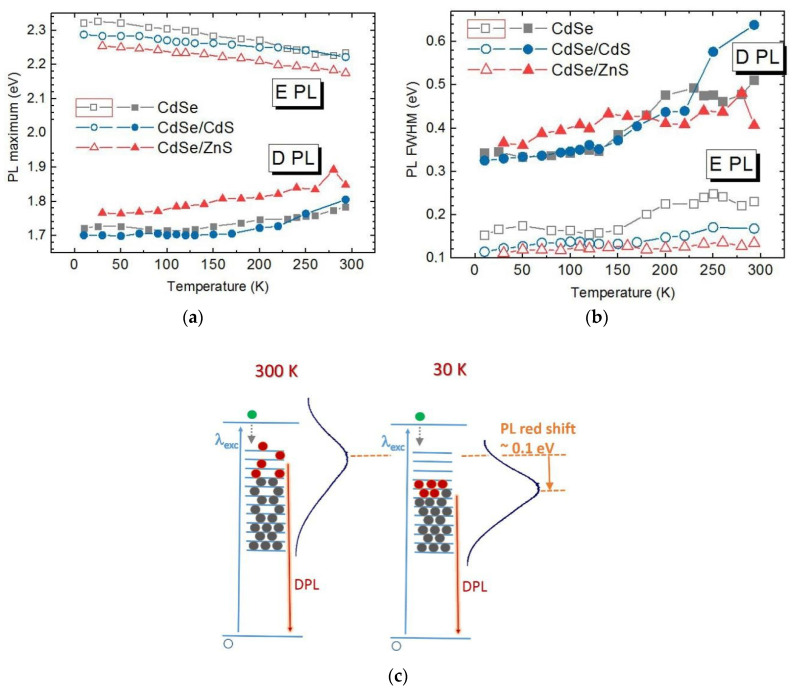
Temperature dependence of the peak position (**a**) and FWHM (**b**) of EPL and DPL bands of CdSe, CdSe, and CdSe/ZnS NCs stabilized with gelatin (λ_exc_ = 441.7 nm, 0.1 W/cm^2^). The schematic in (**c**) illustrates the assumed mechanism behind the observed red shift of the trap-related PL with temperature decrease.

Figure 9 summarizes the temperature dependence of the peak position and FWHM of EPL and DPL of the three samples of NCs in a gelatin matrix discussed above. One more anomalous feature of the DPL band can be seen from this figure. In particular, a pronounced redshift of the DPL band is observed for all samples (Figure 9a), contrary to the well-established PL behavior upon decreasing temperature, which includes blue shift and narrowing of PL bands [55,56]. We attribute this rather anomalous behavior to the suppressed thermal occupation of higher-energy trap states, resulting in a lower average energy of the occupied trap states at lower temperature, and thus a lower energy of the PL maximum. This suggested mechanism is schematically illustrated in Figure 9c. We note, that the observed anomalous temperature dependence of the DPL FWHM is a very pronounced indication of the role of the redistribution of charges over trap states in the PL properties of the NCs studied here.

Another important conclusion made from the red shift of the DPL upon temperature decrease is that the radiative recombination resulting in DPL occurs between deeply trapped electrons and holes in the band states or at shallow traps. This picture is in accordance with the band diagram suggested above (Figure 3) based on the PL photoenhancement data. If holes were the deeply trapped carriers involved in DPL emission, a blue shift would be observed for the band maximum. These findings are another illustration of the fact that despite decades of intense investigations of II-VI NCs PL there is still a lively discussion on whether trapped electrons or trapped holes are responsible for the large Stokes shift of DPL [57]. Therefore, the data obtained in this work can be regarded as a substantial contribution to solving this dilemma.

### 3.4. Time-Dependent PL of NCs Synthesized Ex Situ and Subsequently Introduced into a Polymer Matrix

In order to obtain a more general view of the photoenhancement phenomenon and of the underlying mechanism, we investigated other types of II-VI NCs, which were produced not in situ in aqueous solutions of polymers, but first synthesized using small-molecule ligands/stabilizers and then transferred into a polymer matrix. 

**Figure 10:**
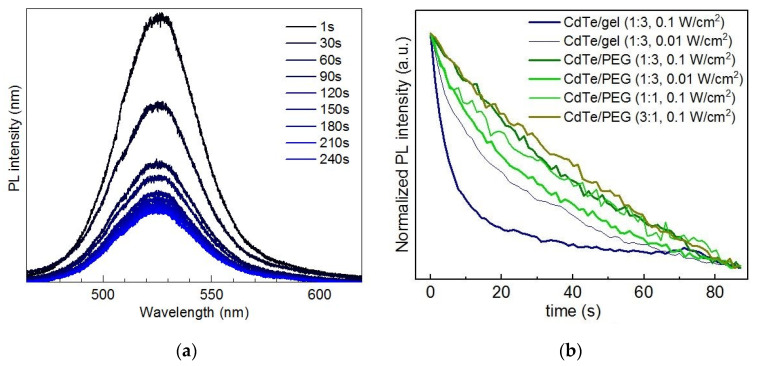
(**a**) Evolution of the representative room-temperature PL spectra (λ_exc_ = 325 nm, 0.1 W/cm^2^) of CdTe/CdS NCs embedded in gelatin. (**b**) Time-dependent behavior of PL intensity (λ_exc_ = 515 nm, power density indicated in the legend) CdTe/CdS NCs embedded in gelatin and PEG at different volumetric ratios of NC:polymer.

For CdTe NCs synthesized using thioglycolic acid (TGA) as a stabilizer and subsequently introduced into a polymer, we observed a noticeable degradation of the PL intensity (Figure 10) that occurred on the same timescale and excitation power as the photoenhancement was observed above for polymer-stabilized CdSe and core/shell NCs (see Section 3.1, Figure 2 and Figure 4). Even though there was a trend toward saturation of the PL intensity at longer time scales (hundreds of seconds to tens of minutes), which was inversely proportional to the excitation power (Figure 10b), no indication of subsequent intensity enhancement was observed. In Figure 10 the results are presented for gelatin and polyethylene glycol (PEG), but qualitatively similar results were obtained for PVA and polyvinyl pyrrolidone (PVP). Nevertheless, no severe structural degradation can be concluded as evidenced by the constant position of the EPL peak (DPL was not observed for this sort of NCs). This is an important implication of the polymer matrices especially for CdTe NCs, because compared to its sulfide and selenide counterparts telluride compounds are generally more sensitive to photodegradation [58], but even a gelatin matrix ensures sufficient protection of the NCs against photooxidation. For instance, strong spectral changes were observed in both optical absorption and PL of aqueous CdTe NC solutions under prolonged illumination even with the UV-component of sun irradiation [59].

**Figure 11:**
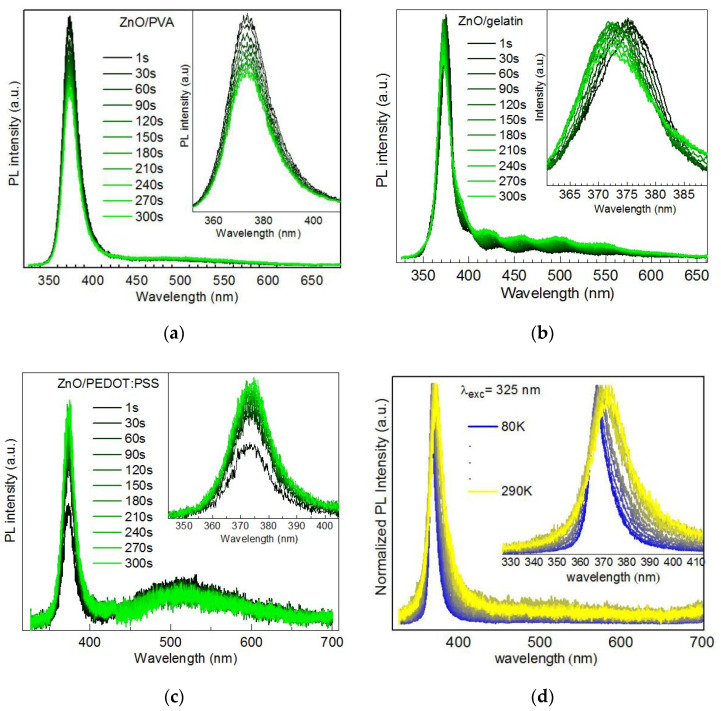
Evolution of the room-temperature PL spectra (λ_exc_ = 325 nm, 0.1 W/cm^2^) of ZnO NCs in a matrix of PVA (**a**), gelatin (**b**), and PEDOT:PSS (**c**). The temperature dependence of PL of ZnO NCs in PEDOT:PSS is shown in (**d**). The waviness of the PL curve in (**b**) is due to interference in the polymer film.

A distinct behavior was observed for ZnO NCs synthesized ex situ using no additional stabilizers except zinc acetate that served as a source of zinc in the synthesis and tetraethyl ammonium. This was the only type of NCs that revealed qualitatively different behavior in different polymers, and different from the other NCs. In particular, for ZnO NCs embedded into a PVA matrix, a slight decrease of the PL intensity with illumination time was observed (Figure 11a). When embedded into a gelatin matrix, the EPL band revealed a noticeable blue shift at a rather unchanged PL intensity (Figure 11b). When using conductive poly(3,4-ethylenedioxythiophene) polystyrene sulfonate (PEDOT:PSS), a certain enhancement of the PL intensity was observed at very early time of illumination, followed by a quick saturation of the PL intensity, with no measurable shift of the PL peak position (Figure 11c). 

The investigation of the temperature dependence of the PL spectra of ZnO NCs in a PEDOT:PSS matrix revealed its ordinary behavior consisting of a blue shift and narrowing at lower temperature (Figure 11d). Therefore, as for the CdSe based series of samples analyzed above, we can conclude that the illumination with the UV (or other) laser wavelength of the chosen laser power does not cause any noticeable structural modifications of the NCs themselves or the surrounding matrix or the NC/polymer interface. The observed evolution of the PL intensity with illumination time is predominantly an effect of accumulation and redistribution of the charge carriers either between different types of electronic states within the NC (including its surface) or between the NC and medium (polymer). 

### 3.5. Excitation Power Dependence of the PL Spectra of NCs 

**Figure 12:**
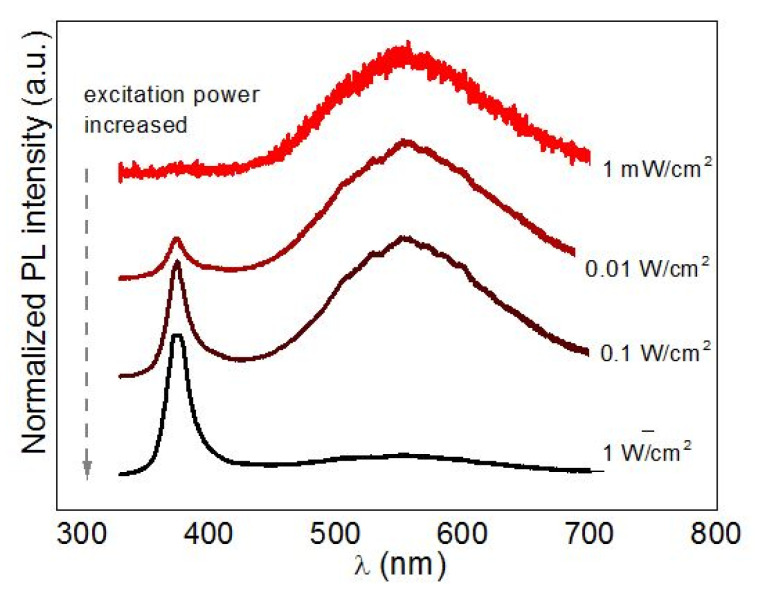
Evolution of the representative room-temperature PL spectra of ZnO NCs in solution as a function of the laser excitation power (λ_exc_ = 325 nm).

One more experiment that proves the determinant role of the saturation of trap states in the PL photoenhancement of NCs in polymer matrices is the dependence of the PL spectra on the excitation power. Figure 12 shows a representative set of spectra for ZnO NCs. The spectra are normalized for convenience of showing clearly the change in the EPL/DPL intensity ratio. One can see that with an increase of the excitation power, along with the expected increase of absolute PL intensity (seen in the normalized spectra as improved signal-to-noise ratio), the EPL/DPL intensity ratio increases dramatically. As in the experiment with time-dependence of PL spectra discussed above, no change in EPL peak position and FWHM was observed. Moreover, the observed intensity redistribution of the EPL and DPL was completely reversible. The latter two facts undoubtedly prove that the observed spectral changes are not due to structural changes in the NCs, induced by the exciting laser light, but due to the redistribution of the charge carriers over energy levels of the trap states. A thermal distribution of the charge carriers over the energy levels of radiative traps at room temperature is apparently sufficient for the DPL position not to be affected (Figure 12) by saturation of the non-radiative states.

One of the key issues discussed regarding photoenhancement and photodarkening is whether it is due to an increased number of “bright” (i.e., emissive) NCs or due to an increased PL quantum yield (QY) of already emitting ones. For NC/polymer samples studied here, we assume that the former mechanism does not take place, because the photoinduced changes in PL intensity are not accompanied by a peak shift or a change in the FWHM of the excitonic band. The latter spectral stability of the excitonic PL band is a solid argument to exclude photooxidation (photocorrosion) as a reason of enhanced PL intensity. In some studies [24], the removal of surface SeO_2_ was suggested for the observed changes in PL intensity of CdSe-based NCs. The role of oxygen in the photo-induced process involving QDs is potentially important and was invoked in many reports on PL photoenhancement. In the present NC/polymer composites, the role of oxygen does not seem to be decisive, because we did not observe a stimulation of the photoenhancement in water compared to films in air and did not observe a significant weakening of the effect in vacuum as compared to films in air. Even though the possibility of the formation of elemental Se on the surface of CdSe NCs without polymer was suggested from SERS/TERS studies [60,61], its contribution to the PL spectra of CdSe NCs as a reason of a different behavior as compared to core/shell NCs is unlikely. Se nanoparticles formed in the matrix of the same polymer (e.g., gelatin) were shown to be non-luminescent without additional annealing [62].

Even though we relate the observed PL enhancement of NCs-in-polymer with filling of the trap states with charges, the trapped charges do not cause spectral changes. In particular, previously we observed the suppression of the DPL and rise of an additional EPL band in photo-charged ZnO NCs in ethanol, where the photocharged electrons occupied partially the conduction band levels (Burstein-Moss effect) [63]. A red shift of EPL in many single-QD studies was observed under continuous illumination and explained by the Stark effect due to locally trapped charges [64]. 

Photoinduced PL quenching of green-emitting NCs and its stability for red-emitting colloidal CdTe-TGA NCs embedded in a SiO_2_ matrix observed in [65] was interpreted by the authors by a different state of the NC surface. However, this effect can also arise from a much thicker CdS shell on red-emitting NCs, which were synthesized for a much longer time (100 h) as compared to the green ones (1.5 h). Therefore, the photoenhancement observed in this work is distinct from previous experiments of NCs in polymer matrix, which showed either brightening and spectral changes [24,25,34] or darkening and no spectral changes [34].

**Figure 13:**
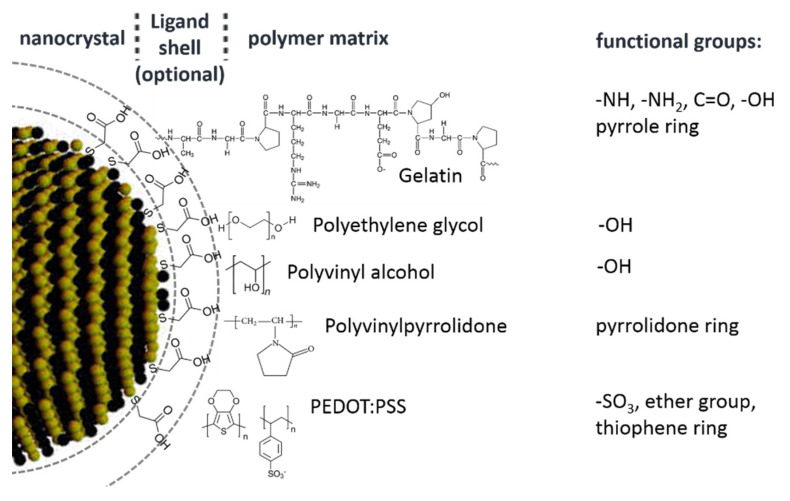
Schematic representation of the NC/polymer interface, including an optional ligand shell formed in case of ex situ synthesized NCs (see text for details and discussion).

An enhancement of the PL QY of CdSe QDs when photoactivated above the band gap in the presence of polymers such as poly(dimethyl siloxane) (PDMS), PVP, and poly(butadiene) (PBD) was reported in [59]. Contrary to our work, in this report CdSe NCs were synthesized not by an aqueous route but by organic synthesis, using trioctylphosphine (TOP) and trictlyphospine oxide (TOPO) as stabilizers, and then transferred into polymers. The lack of a direct NC/polymer interface may be the reason for the lack of spectral stability of the PL band in the latter work—a blue shift as large as 20–30 nm was observed at a timescale comparable to that in our work (tens of seconds to tens of minutes). In our systems with ex situ synthesized CdTe-TGA NCs embedded into several polymers, including PVP also used in Ref. [59], no spectral shift was observed, although we also observe some PL decrease, contrary to PL enhancement for polymer-stabilized NCs (3.1). Therefore, we can assume that the formation of the direct interface of NCs with the polymer may not be the only way of obtaining spectral photostability of the NC PL. In the case of a TGA ligand, its sulphur atom is a part of the NC lattice and, therefore, the NC-ligand assembly is much stronger as compared to the NC-TOPO kept together by coordination bonding. Therefore, TGA-stabilized NCs embedded into a polymer matrix can better withstand photooxidation than the NC-TOPO system. Nevertheless, for the PL photoenhancement to take place, as shown above in this work, NCs directly stabilized with polymer seem to be more efficient. At that, the larger variety of function group in gelatin (Figure 13), as compared to PVA, for instance, can be responsible for stronger photoenhancement in the CdSe/gelatin composite (Figure 2) as compared to CdSe/PVA (Figure 5b). In particular, amine groups of gelatin can play a vital role in this effect, because their bonding to the metal ion on the NC surface was suggested to be responsible for the stability and high PL QY of ultrasmall CdS NC stabilized in situ by polyethylenimine [66].

**Figure 14:**
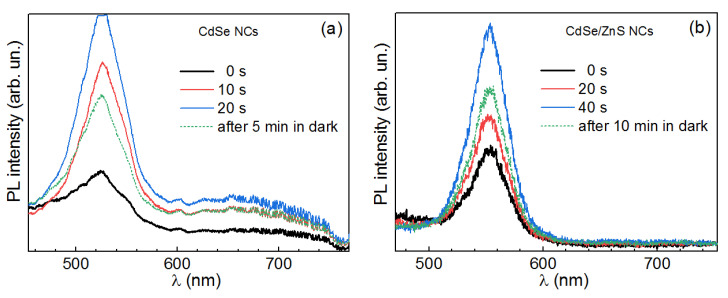
Illustration of the reversibility of the PL photoenhancement for both CdSe (**a**) and CdSe/ZnS NCs (**b**) at λ_exc_ = 325 nm.

If photoannealing of NC defects were the reason of the observed photoenhancement in our study, it would be expected to have a comparable effect on the NC/polymer samples with very close absorptivity of the (same) λ_exc_ by the NCs and polymers, as well as comparable thermal conductivities and other relevant properties. In particular, CdSe-gel, CdSe-PVA, CdTe-gel, etc., would reveal qualitatively similar trends, which is not the case. A more likely reason for the photoenhancement could be an improved crystallinity of the polymer and the NC/polymer interface, as suggested in the DPL photoenhancement study [32] of CdSe/PVA NCs similar to those in our work. In particular, it was suggested that in the NC/PVA composite, the individual functional groups of PVA as well as the fragments of the partially-broken polymer chains can passivate under-coordinated surface atoms of the NCs with the formation of chemical complexes between the –C=O group of the PVA matrix and the Cd^2+^ ions on the NC surface [32,53]. However, if the specific bonding were dominant and common to all NC/polymer samples studied in this work, we would observe more similarity in the photoinduced behavior of different in situ and ex situ embedded NCs in the same polymers, on the one hand, and less similarity between similar NCs embedded in different polymers. Moreover, the PL photoenhancement observed in this work on NC/polymer samples was to a large extent reversible, in the same time scale as the photoenhancement was observed (Figure 14). 

Therefore, at least the reversible part of the photoenhancement is for sure related with the redistribution of charges within the system but not with the irreversible light-induced structural changes in the polymer or in NCs. The remaining (irreversible) part of the PL intensity does not necessarily indicate structural changes in the sample. A part of charges that were led into traps via high-energy states (as a result of photoexcitation) may not leave these states on their own and may require some activation energy, e.g., heating. 

## 4. Conclusions

We have observed a photo-induced enhancement of PL emission of various colloidal NCs embedded in water-soluble polymers. Based on the comparison of results obtained on bare and core/shell NCs, NCs of different compounds (CdSe, CdTe, ZnO), using different embedding polymers, by analyzing temperature and excitation power dependence of the PL spectra, and a certain reversibility of the intensity change, we conclude that the most probable mechanism of the photoenhancement for this sort of systems is the redistribution of the photoexcited charge carriers over the trap states. Contrary to photoenhancement (or photodarkening) commonly observed before as a result of surface photocorrosion or photoannealing of NCs, we do not observe a change in peak position and width of the excitonic PL bands. Therefore, we suggest that the accumulation of photo-excited charges in trap states, leading to a saturation of the latter, plays a key role in the subsequent enhancement of the radiative recombination. In particular, with increasing the illumination time at a constant laser power or with increasing the laser power at a constant time, a (partial) saturation of the non-radiative states results in an enhanced radiative recombination as a whole. Within this enhancement, depending on the density of the radiative trap states (responsible for DPL band), the ratio between the recombination rates of EPL and DPL, efficiency of the re-trapping to band states, properties of the NC environment, and probably other factors, the enhancement will be stronger either for EPL or for DPL. Although the contribution of light-induced structural changes (crystallization, curing of defects etc.) cannot be fully excluded, establishing its quantitative portion is a scope of a separate study.
